# Supplementary material for: Developmental activities of the complement pathway in migrating neurons
Source: Nat Commun. 2017 May 2;8:15096. doi: 10.1038/ncomms15096 (PMC5418580; doi:10.1038/ncomms15096)
Supplement: Supplementary Information — Supplementary Figures. [file ncomms15096-s1.pdf]

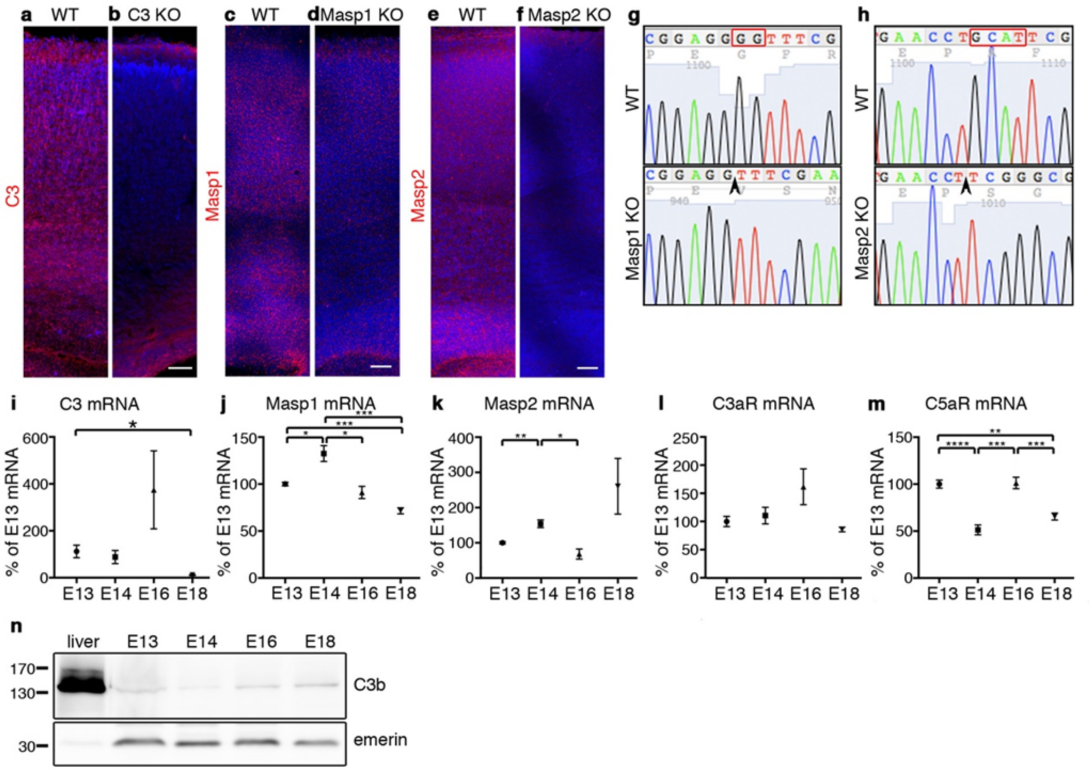

Supplementary figure 1: Expression of complement in the developing brain. (a-f) Immunostainings of embryonic brain slices (E18) of littermate wildtype (a,c,e) and either *C3* knockout (b), *Masp1* knockout (d) or *Masp2* knockout (f) using anti-C3 antibodies (a-b), anti-MASP1 antibodies (c-d) or anti-MASP2 antibodies (e-f). The immunostaining signal is markedly reduced in the corresponding knockout brain sections. Scale bars are 50 µm. (g-h) Sequences derived from *Masp1* (g) and *Masp2* (h) CRISPR-Cas9 gene edited embryos show a two and four base-pair deletion, respectively, leading to frame-shift mutations and premature termination. (i-m) Real-time qRT-PCR showing the relative mRNA expression pattern of *C3* (i), *Masp1* (j), *Masp2* (k), *C3aR* (l), *C5aR* (m). For each time-point (E13, E14, E16, E18) cortices from 3 different embryos were used. Expression data was normalized to the expression level of the ribosomal protein *29rps*. The expression in each day is presented as % of the relative expression observed at E13 of each gene. One-way ANOVA, Turkey HSD. *, $p<0.05$; **, $p<0.01$; ***, $p<0.001$; ****, $p<0.0001$. (n) C3 is proteolytically processed in developing cortex. Levels of activated C3 (C3b) were checked by western blot analysis in cortices from E13, E14, E16 and E18 wild type brains, liver lysate was used as a positive control. Uncropped western blots are shown in Supplementary figure 8.

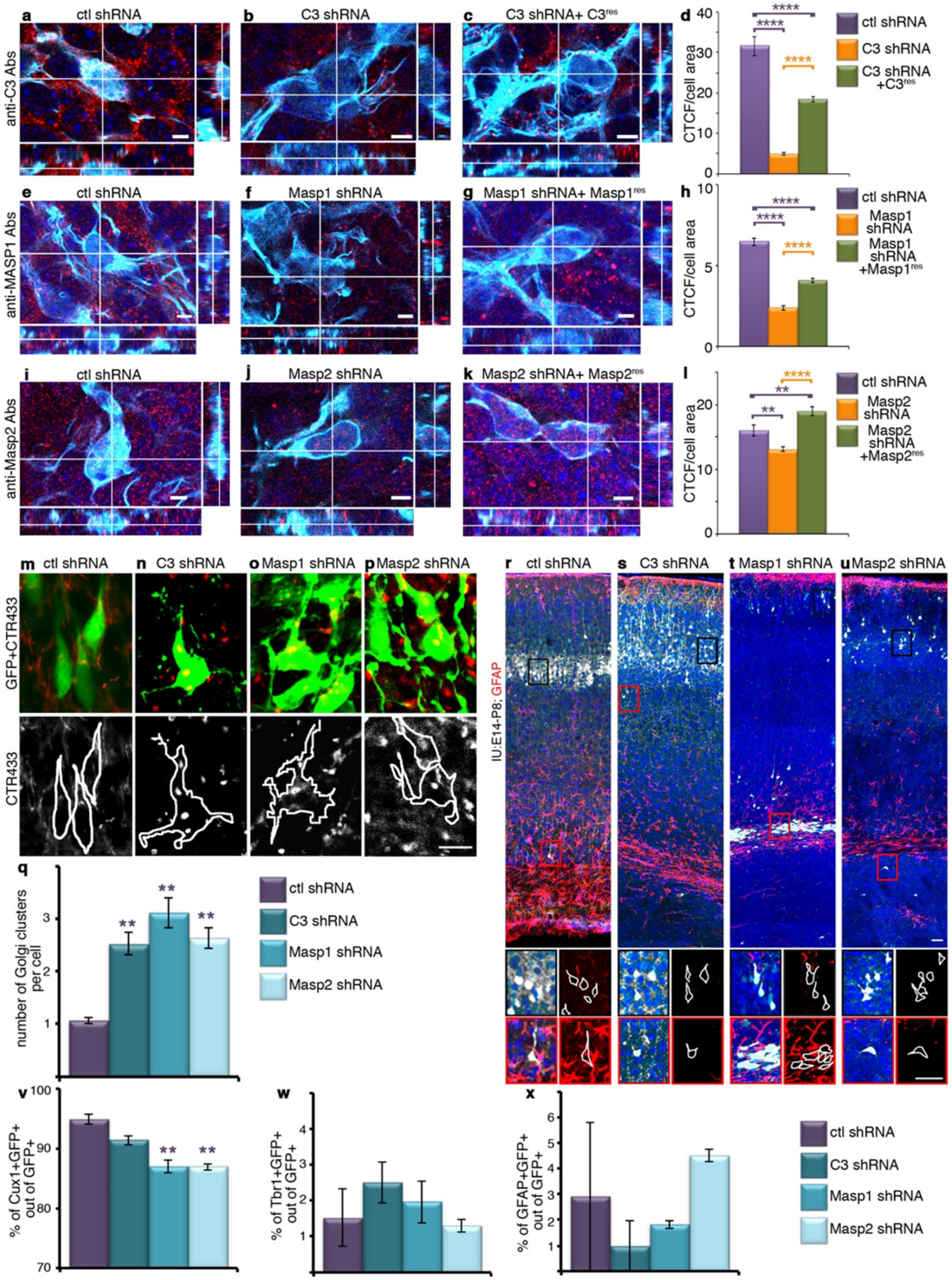

Supplementary figure 2: Validation of shRNA. (a-l) Embryonic mouse brains were *in utero* electroporated (E14-E16) with *C3* shRNA (b), *Masp1* shRNA (f) or *Masp2* shRNA (j) either alone or in combination with relevant expression construct resistant to the shRNA (c,g,k respectively). Control shRNA (a,e,i) served as a reference. Lifeact-GFP

was used to mark the cell periphery. Sections were immunostained with anti-C3 (a-c), anti-MASP1 (e-g), and anti-MASP2 (i-k) antibodies. High-magnification 3D reconstructions of individual neurons performed using confocal microscopy and IMARIS software are shown for each treatment. The scale bars are 3 μm. The corrected total cell fluorescence (CTCF) normalized to the cell area was analyzed using ImageJ (d,h,l, n=20 for each condition). *, $p<0.05$; **, $p<0.01$; ***, $p<0.001$; ****, $p<0.0001$. (m-q) Neuronal morphology and Golgi analysis in *C3*, *Masp1* and *Masp2* knockdown treatments. *C3* shRNA (n), *Masp1* shRNA (o) or *Masp2* shRNA (p) electroporated brain sections (E14-E18) were immunostained with Golgi marker antibodies (CTR433). To observe control shRNA treated cells in the same brain area, the timing of the control experiment was from E14 to E17 (m). Immunostaining of the Golgi are presented together with GFP (top) or with the outlines of neurons (bottom). Fragmented Golgi corresponds to multipolar morphology. The scale bar is 10μm. (q) Quantification of the number of Golgi clusters per cell: control, *C3*, *Masp1* or *Masp2* shRNA treated cells (n=20, one-way ANOVA, Turkey HSD test). (r-u) Brains electroporated *in utero* on E14 with control shRNA (r), *C3* shRNA (s), *Masp1* shRNA (t) or *Masp2* shRNA (u) were immunostained at postnatal day 8 (P8) with anti-GFAP antibodies. Black and red boxes in the pictures show the positions of the box enlargements underneath each slice. The immunostainings for the enlarged areas are shown together with GFP or with the outline of the GFP-positive cells. The scale bar is 50 μm. (v-x) Quantification of relative amount of CUX1-positive (v, fig 2f-i, one-way ANOVA, Turkey HSD test), TBR1-positive (w, fig 2j-m, one-way ANOVA) or GFAP-positive (x, r-u, one-way ANOVA) electroporated cells for *C3*, *Masp1* and *Masp2* knock-down (E14-P8) treatments.

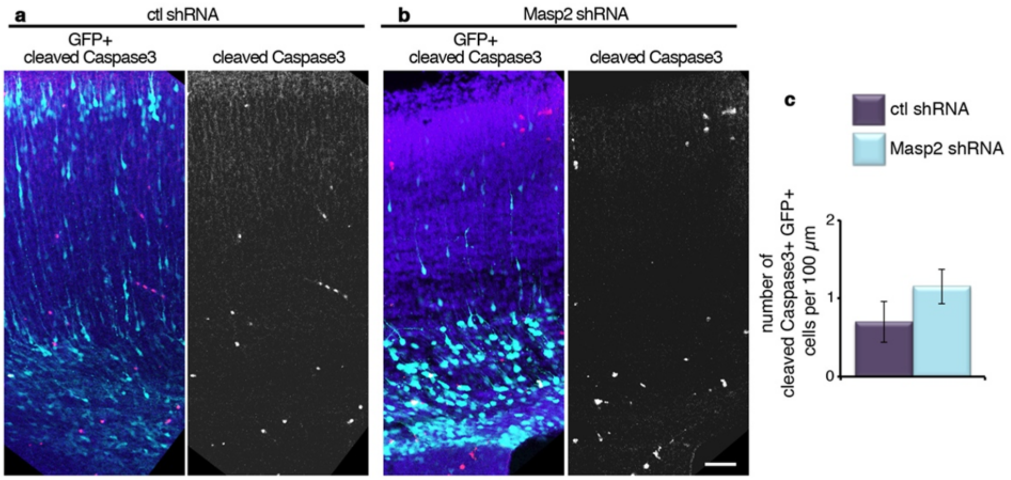

Supplementary figure 3. Brains were in utero electroporated (E14-E18) with control (a) or *Masp2* shRNA (b). The immunostainings with anti-cleaved Caspase3 antibodies were performed. The immunostainings are shown together with GFP (left) or alone (right). GFP-positive/cleaved Caspase3-positive cells were counted and presented in a graph (c) as number of double positive cells per 100 μm width of cortex. The scale bar is 50 μm.

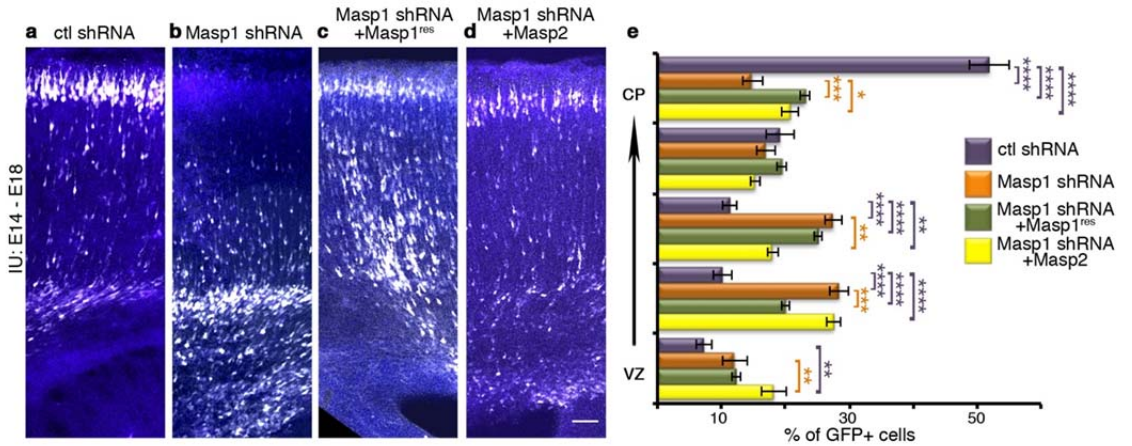

Supplementary figure 4. *Masp1* knockdown and rescue. Brains were electroporated *in utero* (E14-E18) with control shRNA (a, n=4), *Masp1* shRNA (b, n=5), *Masp1* shRNA together with *Masp1* resistant to the shRNA (c, n=4) or *Masp1* shRNA together with *Masp2* (d, n=4). The relative positioning of electroporated cells across the width of the cortex is shown for all the treatments (e) in 5 bins (from the VZ to the CP). Comparison to the WT is shown in violet. Comparison to *Masp1* shRNA condition is shown in orange. *, $p<0.05$; **, $p<0.01$; ***, $p<0.001$; ****, $p<0.0001$.

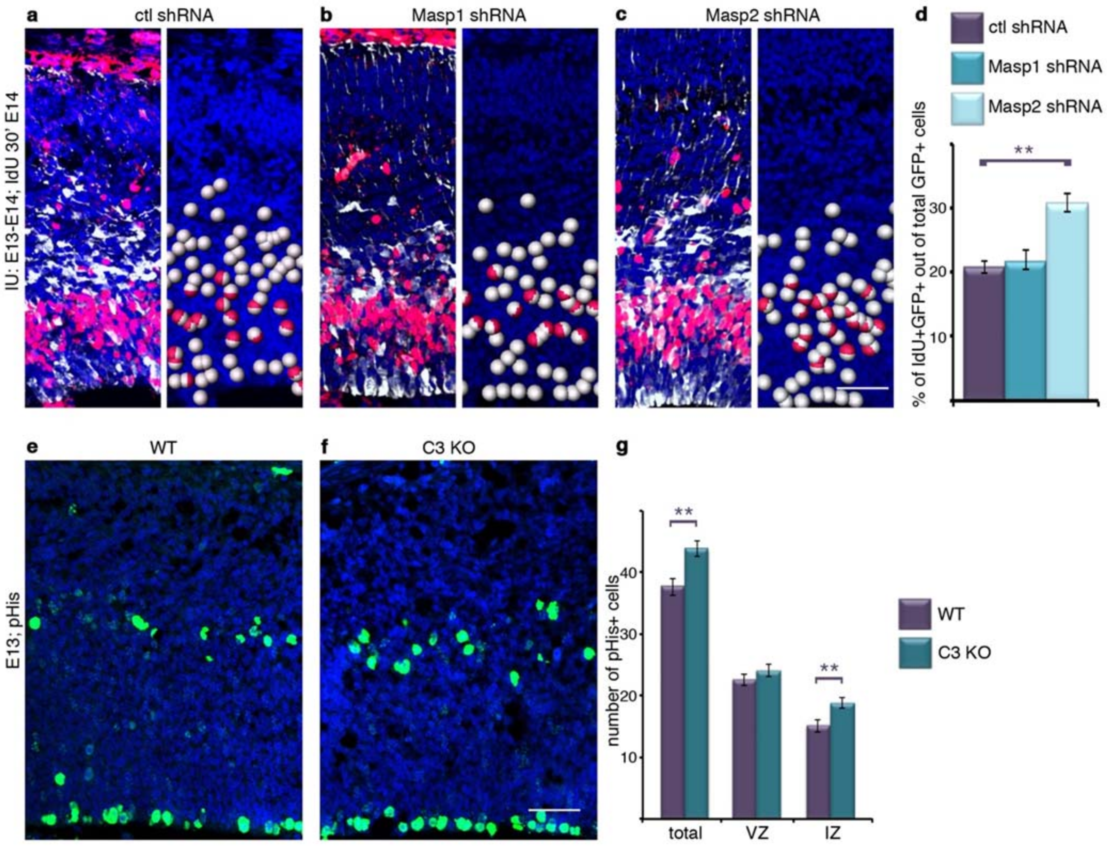

Supplementary figure 5: Proliferation analysis. (a-d) Brains were *in utero* electroporated with control shRNA (a, n=4), *Masp1* shRNA (b, n=4) or *Masp2* shRNA (c, n=4) at E13 and at E14 were treated with IdU for 30 minutes. The brains were cryosectioned and immunostained with anti-IdU antibodies. GFP labeled the electroporated cells. IMARIS software was used to count the total GFP-positive cells and the double GFP- and IdU-positive cells within slices of the same size. The IMARIS visualization is presented on the right side of each slice. The relative proportion of double-positive cells in relation to the total number of GFP-positive cells was calculated, only *Masp2* shRNA treatment significantly differed from the control (one-way ANOVA, Turkey HSD, **, $p<0.01$). (d). (e-g) Brains slices of E13 *C3* KO (e, n=3) and littermate WT (f, n=3) immunostained with anti-pHis3 antibodies. The number of pHis3-positive cells in the VZ and in the IZ was counted (g) in identical areas of the cortices (420 μm in length). The scale bars are 50 μm.

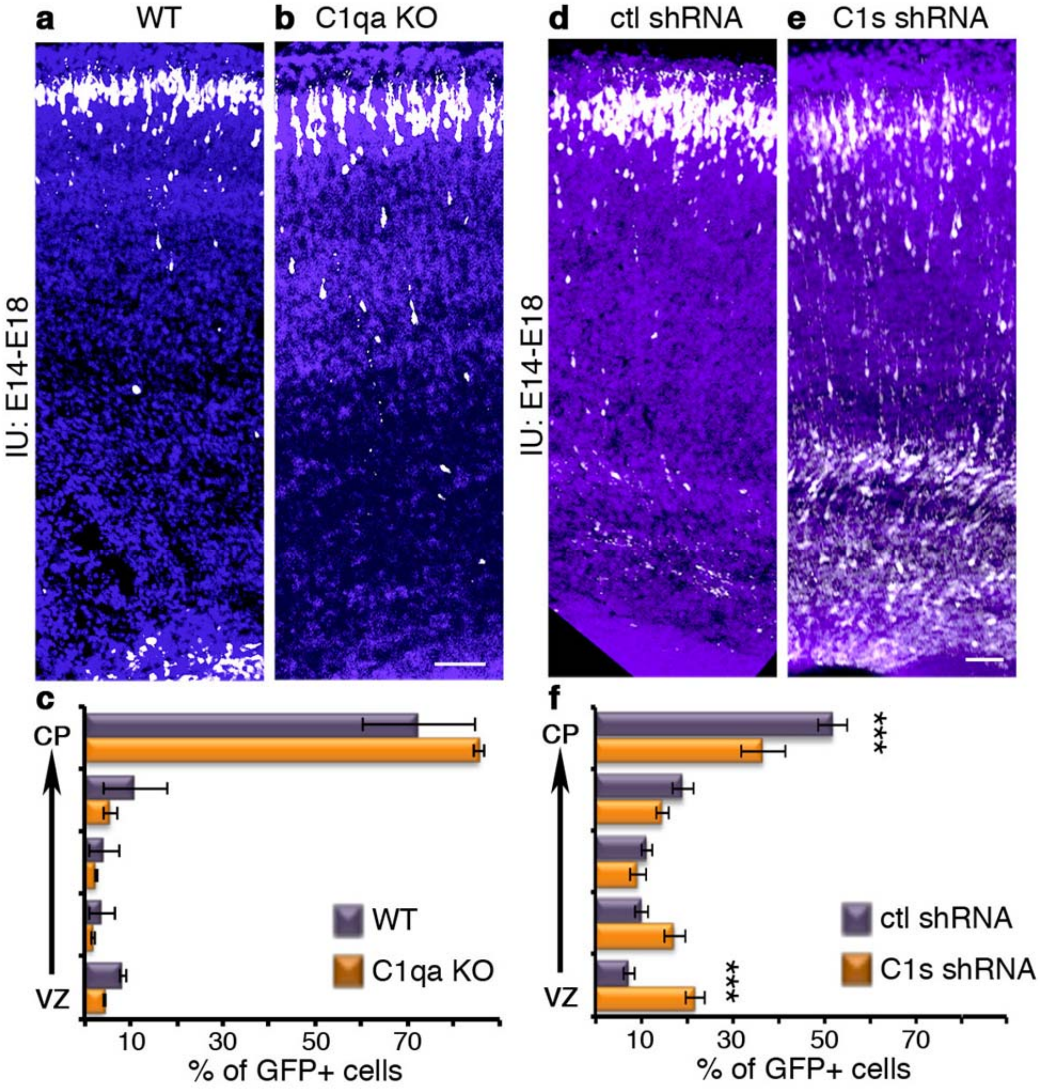

Supplementary figure 6. The possible role of classical pathway proteins. (a-c) *C1qa* KO mice exhibit normal neuronal migration. WT (a, n=3) or *C1qa* KO (b, n=3) embryos were *in utero* electroporated with a GFP expression plasmid at E14 and brains were fixed at E18. The position of the labeled cells is identical, quantified and analyzed (c). (d-f) *C1s* has minor effects on neuronal migration. Brains were electroporated *in utero* (E14-E18) with control shRNA (d) or *C1s* shRNA (e). The position of the electroporated cells was quantified and analyzed (f). The scale bars are 50 µm. ***, $p<0.001$.

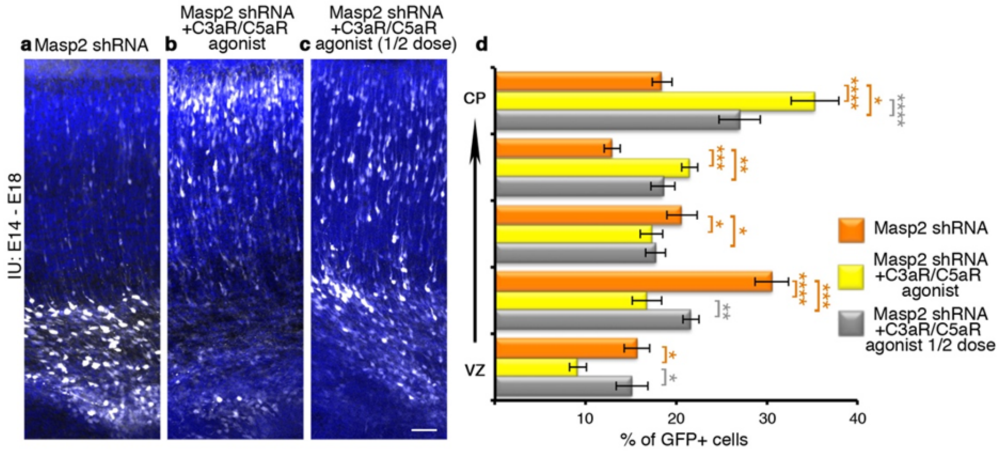

Supplementary figure 7. Dose-dependant effect of C3aR/C5aR agonist. Brains were electroporated *in utero* with *Masp2* shRNA (a) alone or together two different concentrations of C3aR/C5aR agonist (b,c). The relative positioning of electroporated cells across the width of the cortex is shown for all the treatments (d) in 5 bins (from the VZ to the CP). Comparison to *Masp1* shRNA condition is shown in orange. Comparison between two conditions with different agonist concentrations is shown in grey. *, $p<0.05$; **, $p<0.01$; ***, $p<0.001$; ****, $p<0.0001$.

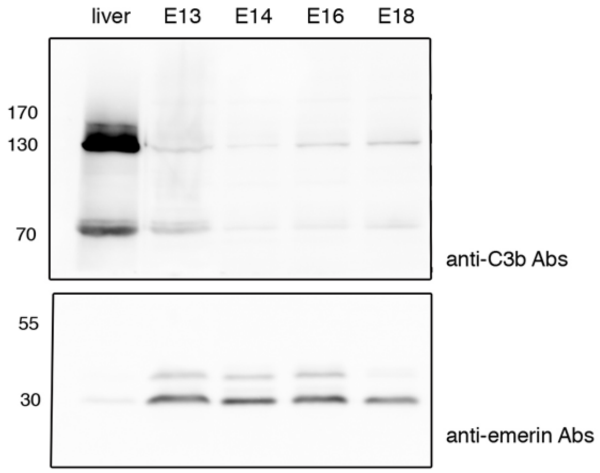

Supplementary figure 8. Full-size scans of the Western blot (Supplementary fig. 1n). Protein extracts from mouse liver, and cortices from mouse embryonic brains (E13,14,16 and 18), were separated on an SDS-PAGE gel and transferred to a membrane. The membrane was cut to two. The top half was incubated with anti-C3b antibodies and the lower half was incubated with anti-emerin antibodies.
